# Health-Promoting and Therapeutic Attributes of Milk-Derived Bioactive Peptides

**DOI:** 10.3390/nu14153001

**Published:** 2022-07-22

**Authors:** Mrinal Samtiya, Sweta Samtiya, Prarabdh C. Badgujar, Anil Kumar Puniya, Tejpal Dhewa, Rotimi E. Aluko

**Affiliations:** 1 Department of Nutrition Biology, School of Interdisciplinary and Applied Sciences, Central University of Haryana, Mahendergarh 123031, Haryana, India; mrinalsamtiya@gmail.com; 2 Department of Food Science and Technology, National Institute of Food Technology Entrepreneurship and Management, Kundli, Sonipat 131028, Haryana, India; prarabdh.badgujar@gmail.com; 3 Trained Graduate Teacher (TGT) Science, Kendriya Vidyalaya No.2, Jalahalli East, Bengaluru 560014, Karnataka, India; kaushiksweta23@gmail.com; 4 Dairy Microbiology Division, ICAR-National Dairy Research Institute, Karnal 132001, Haryana, India; akpuniya@gmail.com; 5 Department of Food and Human Nutritional Sciences, University of Manitoba, Winnipeg, MB R3T 2N2, Canada

**Keywords:** milk proteins, bioactive peptides, anti-inflammation, anti-diabetic, anti-hypertensive, therapeutic potentials

## Abstract

Milk-derived bioactive peptides (BAPs) possess several potential attributes in terms of therapeutic capacity and their nutritional value. BAPs from milk proteins can be liberated by bacterial fermentation, in vitro enzymatic hydrolysis, food processing, and gastrointestinal digestion. Previous evidence suggested that milk protein-derived BAPs have numerous health-beneficial characteristics, including anti-cancerous activity, anti-microbial activity, anti-oxidative, anti-hypertensive, lipid-lowering, anti-diabetic, and anti-osteogenic. In this literature overview, we briefly discussed the production of milk protein-derived BAPs and their mechanisms of action. Milk protein-derived BAPs are gaining much interest worldwide due to their immense potential as health-promoting agents. These BAPs are now used to formulate products sold in the market, which reflects their safety as natural compounds. However, enhanced commercialization of milk protein-derived BAPs depends on knowledge of their particular functions/attributes and safety confirmation using human intervention trials. We have summarized the therapeutic potentials of these BAPs based on data from in vivo and in vitro studies.

## 1. Introduction

Milk is considered a vital part of the diet of several populations, with an estimated 600 million people worldwide as regular consumers [1]. Buffaloes and dairy cows are the primary sources of commercial milk in addition to minor sources such as camels, mare, goat, donkey, sheep, mithun, yak, etc. [2]. In 2019, the global dairy yield (cow milk 81%, buffalo milk 15%, and camel, sheep, and goat combined 4%) surged by approximately 1.3% to nearly 852 million tons (Mt). In contrast, the yield increased by 4.2% to nearly 192 Mt in India, the world’s leading milk-producing country [3]. Epidemiological findings have suggested that milk consumption and the inclusion of its products in the human diet can lead to a decline in the frequency of metabolic disorders [4]. This is because milk proteins contain significant contents of bioactive peptides (BAPs) with several vital health-promoting properties like anti-oxidative, antithrombotic, anti-hypertensive, anti-microbial, immunomodulatory, and infrequently, multifunctional activity [5]. Amino acid valine has (89 Da) the lowest molecular weight (MW), and tryptophan has (204 Da) the highest MW.

BAPs are unambiguous fragments of parent proteins and are smaller than 6 kDa in terms of MW with an average length of 2–20 amino acids. Studies suggest that BAPs consisting of 2–6 amino acids are readily assimilated compared to free amino acids and longer-chain proteins. Once absorbed, they facilitate biological activities in various tissues as they transit through the body [6,7]. BAPs that are encrypted in the primary sequences of proteins can be released by the actions of exogenous and endogenous proteolytic enzymes or microbial fermentation or during food processing. Although enzymatic hydrolysis is the most common approach to generating BAPs from whole food proteins, fermentation is primarily relevant to the products that naturally contain precursor proteins such as milk, soy milk, and other aqueous products. After protein hydrolysis, peptides need to be separated, purified, and characterized using empirical approaches, and finally, obtained peptides are tested for the targeted bioactivities using in vitro, in vivo, and human studies [8,9]. A significant impact has been observed on the host epigenome by the functioning of bioactive compounds produced due to microbial activities during fermentation [10]. These compact BAPs have high bioavailability, low molecular weight, and flexible molecular structure, which facilitate interactions with various receptors in vitro as well as inside the human body [4]. Considering their potential to block enzymatic activities of α-amylase, dipeptidyl peptidase IV (DPP-IV), and α-glucosidase, BAPs can act as messengers against type 2 diabetes. BAPs can also induce an anti-hypertensive effect as they have an affinity for the angiotensin-converting enzyme (ACE), thus competing for substrate binding. BAPs can downregulate cholesterol synthesis and metabolism pathways to produce a hypocholesterolemic effect [11]. In addition, a recent study reviewed the potential of milk-derived BAPs to modulate the gut microbiome, which leads to regulating gut-brain functioning, gut health, and immune system activity [12]. Several studies have been published on milk-derived bioactive compounds, although they are primarily related to the identification, characterization, and curative utilization of a functional food [13,14,15]. This review provides the current status of milk-derived BAPs in managing lifestyle disorders such as type 2 diabetes, hypertension, obesity, cancer, inflammation, bone health, and hypercholesterolemia and their possible mechanism of action. Although, it also contains major milk protein compositions in different species, primary BAPs’ production methods, their purifications and identification methods, use in dairy products application, and the necessity of BAPs’ safety. There is a dearth regarding updated reviews which contain all these sections; here, we cover all the possible parts of milk-derived peptides and describe them critically, which make it different from other reviews. 

## 2. Brief Comparison of Milk and Milk Proteins Composition

For all mammals, milk is considered a good and primary source of nutrients. The major milk constituents, such as proteins, fats, carbohydrates, vitamins, and minerals, fulfill an organism’s basic nutritional requirements [16]. Table 1 shows the major milk composition in the different species. 

Dairy milk primarily contains two varieties of proteins named casein (β, κ, αs1, αs2), which consists of about 80% of the total milk proteins and 20% of whey proteins such as α-lactalbumin, β-lactoglobulin, bovine serum albumin, and immunoglobulins [21,22]. Table 2 shows the major proteins present in the milk of different species. 

However, the milk’s minor and major constituents vary depending on genetic, nutritional, and environmental factors across different animal species. The suitability of milk as a primary substance for several dairy products, its physicochemical and organoleptic characteristics, and its nutritive value are determined by the composition [25]. Furthermore, non-ruminant and ruminant milk are usually different, depending on their constituents. For example, ruminant milk is defined by a high level of total solids along with higher protein, fat, and ash contents [26,27]. Ruminant milk also contains casein as the primary protein fraction; however, the whey protein fraction is greater than the casein fraction in non-ruminant milk. The ratio of casein protein to whey protein largely differs among various species of mammals [2]. Based on this vast diversity in protein types, milk is a valuable source of BAPs with diverse biological activities.

## 3. Production of Milk-Derived BAPs

**Figure 1:**
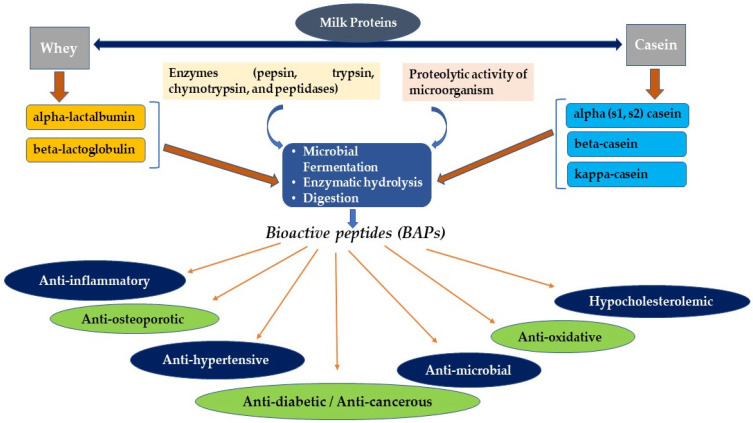
Production of milk-derived BAPs and their possible therapeutic potentials.

BAPs are encoded in the native parent protein amino acid sequence, showing bioactivity only when released from the original root source. BAPs are produced by hydrolysis of proteins utilizing gastrointestinal tract (GIT) digestive enzymes, maturation of food, natural fermentation, fermentation using starter organisms, or food-grade microbial enzymes. In addition to the breakdown of food in the biotic system, GIT enzymes such as trypsin, pepsin, peptidases, and chymotrypsin are tools in BAPs’ production [28,29,30]. Even though BAPs have been obtained conventionally using enzymes extracted from natural sources, several attempts have been made to mass-produce BAPs using recombinant DNA technology [31,32]. Furthermore, other methods, such as the whole expression in the host cells using recombinant DNA, solvent extraction, and peptide synthesis using single amino acids, can be used to obtain BAPs [33]. Figure 1 shows the methods of production of BAPs derived from milk proteins and their possible therapeutic uses.

### 3.1. Fermentation and Enzymatic Hydrolysis Are the Two Most Common Methods Widely Used to Produce BAPs

#### 3.1.1. Fermentation

Fermentation is considered an economical approach for obtaining BAPs. Yeast or various bacteria have been utilized to hydrolyze or ferment proteins into peptides. Microorganisms such as the lactic acid bacteria (LAB) are used frequently in the manufacture of fermented dairy products (cheese, cultured milk, yogurt, etc.). During fermentation, the microbial proteolytic enzymes act upon the parent proteins to release peptides [16,34]. The proteolytic systems of LAB have cell envelope proteinase (CEP), which can hydrolyze milk protein into peptides (4 to 30 residues) [34]. Earlier reports have shown the preparation of several BAPs, including immunomodulatory, antioxidative, antimicrobial, and ACE-inhibitory, using microbial proteolysis [21,35,36,37,38]. Various investigators have also reported LAB’s ability, especially lactobacilli, to produce BAPs. A recent study by Gaspar-Pintiliescu et al. [39] reported the potential functional attributes of bioactive peptides produced through fermentation of *Candida lipolytica* MIUG D67 and *C. lipolytica* MIUG D99 yeast strains. A 10% (*w*/*v*) mixture of bovine colostrum powder containing 2.5% (*w*/*v*) kefir grains was fermented with *C. lipolytica* MIUG D99 (10^4^ cfu/mL) and *C. lipolytica* MIUG D67 (10^4^ cfu/mL) for 48 and 72 h, respectively. Consequently, the fermented colostrum powder was incubated at 1% (*w*/*w*) in water at 4 °C overnight, and then water-soluble extracts (containing peptides) were collected for further use. The extracts were filtered through Ultra centrifugal filters with a 10 kDa nominal molecular weight cut-off membrane. Results found that free radicals scavenging activities of *C. lipolytica* MIUG D99 extract (peptide containing) were more potent than those of *C. lipolytica* MIUG D67 extract. For example, *Lactobacillus casei* Shirota ferments casein, and *Streptococcus* thermophiles produce ACE-inhibitory and antithrombic peptides. *L. helveticus* facilitated casein hydrolysis has been used to produce important ACE-inhibitory peptides like VPP and IPP [40]. Wu et al. [41] have also used the milk fermented with *L. delbrueckii* QS306 to isolate ACE-inhibitory peptides. Tonolo et al. [42] produced a peptide with antioxidant properties using starter organisms like *L. delbrueckii* subspp. Bulgaricus, *L. acidophilus*, and *S. thermophiles*. Fan et al. [43] have also produced various BAPs from casein fermentation using *L. helveticus*. Rubak et al. [44] used the indigenous LAB to generate ACE-inhibitory peptides from fermented milk. These results suggest selective fermentation is an excellent way to generate peptides with biological activities. Therefore, implementing such selective fermentation with better proteolytic strains in the production of fermented products would also improve their functional attributes and accelerate the milk utilization rate. Table 3 shows milk-derived peptides produced using the fermentation method.

#### 3.1.2. Enzymatic Hydrolysis

BAPs obtained using in vitro enzymatic hydrolysis of various food proteins have shown possible uses as health-promoting factors against various conditions related to human health and diseases such as inflammation, cancer, and cardiovascular disorders. Following selecting a suitable food protein, peptides of interest are obtained by performing enzymatic hydrolysis either using single or multiple specific or nonspecific proteases [29]. Appropriate food-grade enzymes are practically employed during hydrolysis to increase the nutritional, functional, and physicochemical properties or decrease the allergenicity of the native protein. The potential of the enzymes to produce various peptides with different bioactivities might depend on several factors such as enzyme-substrate concentration or enzyme type and, to a certain degree, on the reaction conditions such as temperature and pH [16]. After hydrolysis, the soluble portion containing a mixture of peptides is centrifuged to obtain BAPs in the supernatant fraction. To perform further processing and separate or purify the peptides, the supernatant is subjected to membrane ultrafiltration, desalting using gel filtration, cross-flow membrane filtration, and various column chromatography techniques [48]. Digestive enzymes such as chymotrypsin, alcalase, pepsin, thermolysin, and several other enzymes from bacterial and fungal sources have also been utilized either alone or in different combinations to generate BAPs from various proteins [21]. However, pepsin and trypsin are commonly used enzymes since they are involved in the GIT digestion of proteins.

Moreover, most known antimicrobial peptides (AMP) have resulted from trypsin or pepsin hydrolysis [49]. However, trypsin and chymotrypsin-treated milk proteins also yielded peptides with immunomodulatory, antibacterial, opioid, mineral binding, and ACE-inhibitory activities [50,51]. Shazly et al. [52] have used enzymes such as alcalase, trypsin, pepsin, and papain to hydrolyze buffalo casein to generate antioxidant peptides. Fajardo-Espinoza et al. [53] hydrolyzed bovine colostrum whey using pepsin and pancreatin to generate BAPs. Table 4 shows milk-derived peptides produced using the in vitro enzymatic hydrolysis method.

## 4. BAPs’ Purification and Identification

BAPs with unique structural properties obtained during post-translational modifications can be easily identified using omics techniques such as metabolomics, genomics, and proteomics for assessing toxicity and other peptide functions. Additionally, advancements in peptide databases and bioinformatics are important for identifying the actions or functions of such small peptide molecules in different organisms [57,58]. Therefore, knowledge of the amino acid sequence of BAPs is essential in elucidating structure-function properties and predicting new peptides with novel functions. Generally, crude peptides are obtained by the practices commonly used in the production of BAPs, which requires additional purification processes. This is because the crude peptide preparations consist of a combination of peptides, other by-products of reactions, and residues of reagents [59]. Therefore, various separation techniques have been employed to purify such crude peptides. In one of the primary steps used for crude peptides purification, the protein hydrolysates are subjected to ultrafiltration, which utilizes molecular weight cut-off membranes to separate peptides into fractions of a narrow size range [60]. Subsequently, the ultrafiltration fractions are subjected to additional separation by one or more of the following techniques: ion-exchange chromatography, reversed-phase high-performance liquid chromatography, affinity chromatography, size exclusion chromatography, or capillary electrophoresis. In addition, electrodialysis-ultrafiltration (EDUF) can be performed to separate anionic, cationic, and neutral peptides of specific molecular sizes [61]. This EDUF technique has shown high efficacy in separating and concentrating small molecular-sized BAPs with net charges. Previous works reported that net positively and negatively charged BAPs with low molecular weights (300 to 700 Da) were successfully separated from snow crab by-product hydrolysates using the EDUF technique [62,63]. Different techniques are utilized to verify peptide purity, especially mass spectrometry like fast atom bombardment mass spectrometry, electrospray ionization, or ionization mass spectrometry/matrix-assisted laser desorption [59,64].

For the identification of BAPs, several phases need to be passed, i.e., (1) protein isolation from food sources, (2) extraction, (3) digestion/separation, and (4) identification and quantification. Several methods have been used for the isolation of proteins and peptides. The most frequently used method is the aqueous-based extraction process due to protein’s high stability and solubility in alkaline water [65]. For separation of BAPs, the most frequently used techniques involve a variety of chromatography techniques. In terms of recovery and selectivity, the most powerful technique which is used is affinity chromatography [66]. Size-exclusion chromatography (SEC) is also regularly used to purify multidimensional BAPS’ fractionations. SEC is a liquid chromatographic method in which the sample is injected into the column generally made of silica, moved through the column using an aqueous solution, and finally separated by pore permeation differentiation. SEC provides an easy and rapid detail of the molecular weight of peptides. To identify BAPs’ sequence, various techniques are used connected with mass spectroscopy. Due to high sensitivity and accuracy, chromatographic methods, mainly LC-MS/MS techniques, have been used for the last decade because of their high sensitivity accuracy. MS is a method (analytical) that gives essential data of targeted analytes in a sample related to its concentration and structure after its conversion to ions [67]. Mass spectroscopy (MS) uses high-energy electrons to ionize the analytes, which causes molecular fragmentation that further passes via the mass-to-charge analyzer followed by detection as a function of mass-to-charge or mass-to-time ratio (*m*/*z*) [68]. MS methods are generally used to analyze peptides using matrix-assisted laser desorption/ionization (MALDI) and electrospray ionization (ESI), working through the ionization of analytes. ESI transfers the initial solution ions into the gas stage using electrical energy before its detection with MS. ESI-MS is a highly reliable, robust, and sensitive technique to detect the quantities of multiple compounds [69]. 

### Advantage of AAs Identification

Amino acids (AAs) composition identification plays a crucial role in predicting the specific attributes of that peptide. For example, leucine at the *N*-terminal position of peptide had shown immense activity in relation to ACE inhibition, as reported by Wu et al. [70]. A recent study by Sonklin et al. [71] reported that peptide LRLESF had the most potent antihypertensive activity due to leucine’s synergistic effect at the *N*-terminal and phenylalanine at the *C*-terminal. Results of the study concluded that amino acid positions are a significant contributor to deciding the ability and potential activity of BAPs. A recent study also reported the enhanced antioxidant potential of the peptide due to aromatic acids such as tryptophan and histidine because of their hydrogen donation ability [72]. Therefore, identifying the amino acid composition is beneficial to selecting the best BAPs to use further as ingredients for functional food product/supplement development.

**Figure 2:**
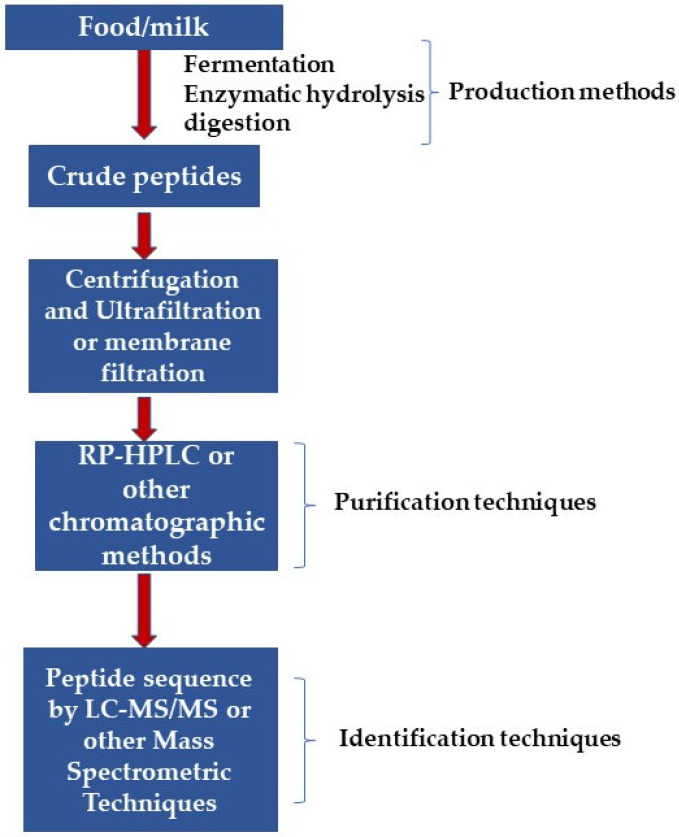
Purification and identification techniques used for milk-derived bioactive peptides.

Figure 2 briefly overviews the purification and identification techniques used for milk-derived BAPs.

## 5. Therapeutic Potentials of Milk-Derived BAPs

BAPs provide essential amino acids and energy, and have been confirmed for their several health promotion characteristics such as osteoprotective, anti-inflammatory, immunomodulatory, anti-microbial, anti-oxidative, hypocholesterolemic, anti-hypertensive, anti-cancerous, and anti-diabetics attributes. To fight against health disorders, pharmacological treatments with drugs have been proven effective but with several unwanted or adverse side effects. In this regard, BAPs derived from milk have been assessed as an adjunct remedy for regulating lifestyle disorders [9,16]. Recently, Li et al. [73] reviewed the mechanism of action and health-promoting attributes of two lactotripeptide (IPP and VPP) against metabolic syndrome, bone health, hypertension ailments, etc. 

### 5.1. Anti-Osteoporotic Effect

Due to the increase in aging populations and life expectancy, it could be expected that the occurrence of osteoporosis will rise considerably in the future [60]. Milk is an immense source of nutrients and minerals such as potassium, magnesium, phosphate, calcium, and proteins, which are crucial for bone development. Casein and whey are the two important proteins found in milk and reported as substrates for the production of BAPs with immense health benefits [74]. Studies confirmed that milk BAPs have the ability to improve bone health through enhancing marker gene (proliferation) expression (i.e., cyclin A and cyclin-dependent kinase 2) [75], by inducing differentiation of osteoblast via Akt signaling cascade [76], and through increasing RUNX2, OCN, ALP, and COL1A1 osteoblast differentiation marker genes expression [77,78]. Previous evidence reported that BAPs derived from milk proteins could be used as active dietary complements to regulate bone-linked ailments such as osteoporosis. In a recent study, VLPVPQK (already validated for its osteoanabolic action), a heptapeptide from milk, was tested, including its 14 novel variants designed in silico. Using calvarial osteoblasts, these variants’ functional attributes were assessed through in vitro assays. Results confirmed that Peptide7 (VLYVPQK) showed the highest response compared to VLPVPQK. Moreover, further results indicated that Peptide7 significantly improved the expression of the osteogenes such as Runx2, Bmp2, Opg, and Osterix [79]. Another study by Reddi et al. [75] confirmed the four BAPs from buffalo casein hydrolysates using pepsin-trypsin hydrolysis, which had an impactful osteoblast proliferation ability. In previous work, researchers investigated the ACE-inhibitory tetrapeptide (YLLF) bone remodeling attributes and antioxidative pentapeptide (YVEEL) whey-derived BAPs using ovariectomized osteoporotic rats. In the study, animals were administered (oral gavage) 50 µg YLLF/kg/day and 500 µg YVEEL/kg/day for 28 consecutive days. Results of the study confirmed that YVEEL showed more osteoprotective properties than YLLF by improving bone formation markers and suppressing inflammation [80].

### 5.2. Anti-Hypertensive

Hypertension is considered one of the foremost causes of death due to cardiovascular-related disorders. As a result of its severe consequences and high occurrence, it is now considered a global health problem. The rising rate of adults facing hypertension increases the necessity for developing new pharmacological treatments [81,82]. Two systems, renin-angiotensin, and kinin nitric oxide, primarily regulate the blood pressure in the body. Renin-angiotensin system controls the angiotensinogen activation and changes it into angiotensin-I through renin proteolytic activity. After that, the angiotensin-I converting enzyme (ACE) cleaved the *C*-terminus histidyl residue of angiotensin-I and converted it to angiotensin-II. Another system is kinin-NO, which mainly takes part in the bradykinin production, which stimulates the reaction to enhance the Ca^2+^ concentration that leads to stimulation of NO production that is a very excellent vasodilator [29]. ACE is a dipeptidase enzyme that cleaves the carboxyl end of the substrate and converts angiotensin-I to the active vasoconstrictor angiotensin-II [83]. A study by Chen et al. [84] assessed the potential of peptides derived from bovine casein against hypertension. Response surface methodology was used with pH 9.01, 61.81 °C, and 6.5% enzyme to substrate ratio, the hydrolysis model showing the best ACE inhibition capability of 85.2%. Further results found two novel peptides, VAPFPE and VLPVPQ, using Q-Exactive LC–MS/MS. Moreover, molecular docking results suggested that these two peptides interact well with the S1 and S2 active sites and Zn (II) of ACE. Previous several studies have shown that milk-derived BAPs possess the capability to regulate hypertension through blood pressure reductions. A recent study used buffalo milk casein to isolate novel peptides after hydrolysis with different enzymes such as chymotrypsin, trypsin, pepsin, and their combinations. Results confirmed that hydrolysates from pepsin-trypsin digestion showed the highest ACE-inhibitory activity. Furthermore, 15 peptides were isolated using the <1 kDa permeate, out of which VLPVPQK is a novel peptide with significant ACE-inhibitory property [85]. A previous systematic review and meta-analysis study reviewed the anti-hypertensive effects of IPP and VPP (lactotripeptides) in Japanese subjects. A total of 18 studies were included in this review, and the study analysis found that IPP/VPP consumptions decrease systolic blood pressure compared to placebo groups. Results of this meta-analysis study suggested that these peptides could efficiently control the blood pressure in the Japanese populations [86]. Previous research investigated the ACE-inhibitory peptides using a couple of complex proteases to hydrolyze bovine milk. Espejo-Carpio et al. [87] reported that goat milk proteins contain encrypted ACE-inhibitory peptides in their primary structure. Therefore, trypsin and subtilisin (individually and in combination) were used to digest goat milk’s whey and casein protein fractions. Results showed that the hydrolyzed casein fraction had the highest ACE-inhibitory property. Furthermore, results confirmed that size exclusion chromatography fraction F2 (<2.3 kDa) possessed the highest activity and concentration of peptides. A long-term clinical study by Jauhiainen et al. [88] evaluated the function of milk drinks with IPP and VPP on augmentation index, endothelial function, and arterial stiffness in human subjects. Eighty-nine hypertensive subjects were supplemented with a low peptide dose (5 mg per day) for twelve weeks and a higher peptide dose (50 mg per day) for the following twelve weeks. At the completion of the second (higher dose) intervention, the augmentation index decreased considerably in the peptide-taking group compared to the placebo. Results confirmed that long-term supplementation of fermented milk (*L. helviticus*) containing IPP and VPP peptides decreases the stiffness (arterial) in terms of augmentation index in subjects (hypertensive). A recent study by Soleymanzadeh et al. [46] reported the ACE-I inhibitory potentials of BAP fractions derived from camel milk fermented by *Leuconostoc lactis* PTCC 1899. Camel milk was fermented for 24 h at 37 °C, and milk’s final bacterial population (*L. lactis*) after inoculation was 10^7^ cfu mL^−1^. Results found that the <3 kDa fraction showed ABTS radical scavenging (1883.39 μM TE mg^−1^ protein) and ACE-inhibitory (IC50 = 1.61 ± 0.18 mg mL^−1^) properties. This fraction was further purified using RP-HPLC followed by identification through MALDI TOF/TOF MS. The most active peptide, i.e., MVPYPQR had ACE-inhibitory IC50 values of 30 μM in addition to antioxidant activity (8933.05 μM TE mg^−1^ peptide) Inhibition of ACE activity is considered an active therapy to reduce hypertension concerns. Still, several factors such as enhanced antioxidative response, nitric oxide-mediated vasodilation, and renin inhibition are assessed in several animals and in vivo trials in relation to the anti-hypertensive properties of BAPs [4,11].

### 5.3. Anti-Hypercholesterolemia

Hypercholesterolemia is one of the considerable health conditions that have been implicated in the pathogenesis and disease progression of cardiovascular disorders. Due to improper diets, fat and cholesterol-rich foods are primarily linked with frequently occurring heart diseases [89]. BAPs are one of the possible strategies that can be used to alleviate hypercholesteremia issues. For instance, a previous study reported the BAPs’ potential to improve hypercholesterolemic conditions might be through the inhibition of lipase activity, inhibition of cholesterol micellar formation, as well as via strong binding to bile acids [90]. It has also been explored that BAPs suppressed the total cholesterol in the serum and inhibited the uptake of cholesterol in monolayer cells. In addition, cholesterol absorption is reduced in the gut due to strong boundation with glycodeoxycholate, deoxytaurocholate, and taurocholate [91]. Previous research studies explored the potential of goat milk casein to improve lipid homeostasis. For the investigation, hypercholesterolemic rats were fed goat milk casein for 30 days. Rats fed with a cholesterol-enriched diet exhibited higher plasma total cholesterol, low-density lipoprotein-cholesterol, and atherogenic indices but lower plasma high-density lipoprotein-cholesterol levels than the standard diet group. This effect was significantly decreased in the cholesterol-enriched diet containing goat milk casein groups, and the high-density lipoprotein-cholesterol levels were also restored [92]. Additionally, a study reported that cow milk-derived BAPs, i.e., lactostatin (IIAEK), had hypocholesterolemic potential in HepG2 human liver cell line, which was more significant in comparison to “sitosterol”, a known anti-hypercholesterolemic drug [93]. Moreover, it has been shown that whey protein-derived BAPs may also have anti-cholesterolemic activity. For example, a study by Nagaoka et al. [94] explored the β-lactoglobulin potential for hypocholesterolemic effects. Results found that rats fed with β-lactoglobulin hydrolysate had reduced liver and serum cholesterol levels. Previous literature information recommended that hypocholesterolemic peptides target exogenous (dietary) cholesterol via hindering absorption from the gastrointestinal tract. Instead of targeting cholesterol, many peptides impede the distribution of bile acids and particularly regulate the metabolism of cholesterol (endogenous) in tissues. BAPs’ hypocholesterolemic ability has been confirmed via the HMG-CoA reductase inhibition, a major cholesterol synthesis enzyme [95]. Furthermore, the study found a novel IIAEK peptide (lactostatin) present in the β-lactoglobulin hydrolysate and was further clarified for its hypocholesterolemic mechanism using in vitro studies. The study utilized human liver cells for screening signal transduction pathways and target genes using this novel IIAEK peptide. Results found that IIAEK regulated intracellular calcium concentration and ERK phosphorylation. Moreover, findings demonstrated the connection of calcium-channel-related MAPK pathways with the IIAEK-mediated cholesterol degradation [93]. A recent study by Jiang et al. [96] reported anti-hypercholesteremic effects (in vitro) of milk-derived peptides, VLPVPQ, VAPFPE, LQPE, and TDVEN via lowering the solubility of micellar cholesterol. Results evaluated that among the peptides, the VLPVPQ peptide showed considerable reduction in the mRNA expression of acetyl-CoA-acetyltransferase 2 (which plays a crucial role in the absorption of cholesterol) using Caco-2 cell line.

### 5.4. Anti-Oxidative

Due to the excessive accumulation of free radicals, oxidative stress occurs in the body. Highly active molecules, such as reactive nitrogen species (RNS) and reactive oxygen species (ROS), are produced during the normal course of cellular metabolism, but reduced intrinsic scavenging capacity can lead to accumulation. Hence, tissue damages occur mainly due to an imbalance between cellular antioxidant capacity and the oxidative systems [97]. Therefore, dietary supplementation with external sources of antioxidants could strengthen cellular capacity for neutralizing toxic free radicals and ameliorating oxidative stress. The antioxidative potential of BAPs is enhanced by factors such as AAs sequence, molecular weight, and their molecular characteristics, including hydrogen donating ability, aromaticity, acid/base character, and hydrophobicity [16]. The presence of AAs (mainly hydrophobic) in the peptides increases their mixing in lipid components and improves availability to free radicals by endorsing antioxidant ability. For example, valine and leucine at *N*-terminal and proline in the peptide sequence stimulate its antioxidant potential [98]. Additionally, peptides containing lysine have an antioxidant ability due to their capacity to chelate Fe^2+^ and Cu^2+^ ions, and Fe^2+^ and Cu^2+^ ions in reduction ability [99,100].

Antioxidant peptides obtained from the enzymatic hydrolysis of milk proteins have improved oxidative stress. For example, previous work reported the antioxidative potential of casein-derived peptide VLPVPQK using rat osteoblastic cells, which were oxidatively stressed by excess hydrogen peroxide (H_2_O_2_). The osteoblastic cells were pretreated with different doses (50–200 ng/mL) of VLPVPQK for 2, 7, or 21 days, each followed by H_2_O_2_ (0.3 mM) treatment for 24 h [101]. Results found that the addition of VLPVPQK led to reduced ROS production, lipid peroxidation, and improved catalase (CAT), superoxide dismutase (SOD), and glutathione peroxidase activities. The most significant property of antioxidative peptides is the presence of hydrophobic AA at the *C*-terminal or *N*-terminal. AAs, for example, Y, W, H, M, C, and K, are the key determinants of BAPs’ antioxidative ability [102,103]. Another study by Sowmya et al. [104] reported the antioxidative potentials of YFYPQL derived from buffalo milk-casein. Results showed that pretreatment of Caco-2 cells with this peptide conferred protection against H_2_O_2_ induced oxidative cell death and hindered ROS production. Furthermore, results confirmed that peptide treatment enriched the anti-oxidative enzymes (glutathione peroxidase, SOD, and CAT) activities by exciting the stress signaling pathway nuclear response factor-2 (Nrf-2). Another study confirmed the antioxidative potentials of YVPR and VPYPQR, which are also milk-derived peptides. Furthermore, study demonstrated that pretreatment with RHPHPHLSFM and VLPVPEK peptides improved the viability of oxidatively stressed Caco-2 cells. These milk-derived peptides are synthesized via solid-phase procedure using automated synthesizer [105].

### 5.5. Anti-Microbial

Anti-microbial capability is a vital hindering property against the growth of diverse pathogenic bacterial strains. This antagonistic property occurs through several routes, including the production of signaling molecules, antioxidants, and bacteriocins. The peptides with antibacterial attributes have variable physiological and biochemical characteristics that promote harmful interactions with target microbes [106]. These peptides comprise hydrophobic and hydrophilic AAs at their terminals, and these are established as the structural motifs used by these peptides to interact with microbes [48]. The possible mechanisms through which these BAPs perform their anti-microbial action via either interrelating with macromolecules inside the microbes or through forming pores in the membrane of microbial cells [107,108]. The vital effective mechanisms through which anti-microbial peptides work are a distortion of the cell membrane by electrostatic interactions, which affect the cell permeability in addition to enhanced inhibition of protein, RNA, and DNA synthesis [16]. For example, α-1 casein-derived peptides are active against a wide variety of Gram-positive bacteria, including *Staphylococcus aureus* [109]. In a recent study by Abu-qatouseh et al. [110], the anti-microbial activity of camel milk-derived peptides was evaluated against *Propionibacterium acnes* using the micro broth dilution method. Results revealed that the peptidoglycan recognition proteins had higher anti-microbial potential than lactoferrin. A comprehensive report identified 207 anti-microbial peptides from milk proteins [111]. Furthermore, the findings suggested that out of the 207 peptides, 177 peptides have unique sequences, and anti-microbial activities were against Gram-negative and Gram-positive bacteria. Methicillin-resistant *Staphylococcus aureus* (MRSA) and *Pseudomonas aeruginosa* are the most virulent pathogens, producing long-lasting and severe human illnesses. A recent study by Abdel-Hamid et al. [112] evaluated the antibacterial attributes of camel milk whey proteins against MRSA and *P. aeruginosa* PAO1. Results showed that camel milk-derived whey protein’s antibacterial activity against MRSA and *P. aeruginosa* PAO1 was improved by papain hydrolysis. Furthermore, the highest antibacterial property was reported for size-exclusion chromatography fraction 2 against MRSA and PAO1 with 0.3125 and 0.156 mg/mL minimal inhibitory concentrations, respectively.

### 5.6. Immunomodulatory/Anti-Inflammatory

Immunomodulatory functions of natural peptides have been recommended as a possible approach to regulating the immune system against immune-linked disorders and infections [113]. Previous studies have reported immunomodulatory/anti-inflammatory attributes of peptides derived from milk proteins. BAPs primarily regulate the inflammation/immune response by controlling the pathways such as peptide transporter 1, NF-κB, (JAK-STAT), and MAPK by controlling the functioning of cytokines such as IL-10, IFN-gamma, TGF-β, etc. [114]. In a recent study by Adams et al. [38], the immunomodulatory properties of peptides produced by the LAB strains *Lactocaseibacillus rhamnosus* R0011 and *Lactobacillus helveticus* R0389 were assessed. In the study, cell-free supernatants obtained from milk fermentation cultures were used as the source of peptides and evaluated for immune regulatory properties, especially inhibition of the production of pro-inflammatory cytokines using human THP-1 monocytes. Results found that specific peptide fractions from the fermented milk could induce interleukin 10 (IL-10) production. IL-10 is an anti-inflammatory cytokine produced by diverse cell types comprising lymphoid and myeloid cells, which mainly plays a significant role in regulating inflammation. It helps alleviate several inflammatory concerns/diseases due to its inhibitory function on pro-inflammatory cytokines [115]. Another study reported the anti-inflammatory attributes of buffalo casein-derived YFYPQL using ex vivo conditions. Specifically, YFYPQL hinders the proliferation of mice splenocytes while improving the phagocytosis activity of peritoneal macrophages. Furthermore, the findings showed a reduction in IFN-γ secretion and enhancement of IL-10 level in the supernatants of splenocytes culture after peptide (buffalo casein derived YFYPQL) treatment [104]. The phagocytosis property of macrophages is the ability to capture and kill the pathogens to protect the host and stimulate innate and adaptive immune actions. Peritoneal macrophages are generally found in the peritoneal cavity and the organs present in the abdomen [116]. Interferon-gamma (IFN-γ) is a pro-inflammatory cytokine that plays a significant role in autoimmune diseases and inflammation [117]. Previous findings by Marcone et al. [118] confirmed the anti-inflammatory potential of bovine milk hydrolysate possessing bioactive peptides. Results found that the milk hydrolysate-treated endothelial cells showed reductions in the expressions of monocyte chemoattractant protein-1 (MCP-1), E-selectin (E-Sel), and vascular cell adhesion protein-1 (VCAM-1), intercellular adhesion molecules-1 (ICAM-1), and IL-8. IL-8 and MCP-1 are inflammatory chemotactic factors that enhance the inflammatory cell/factor expressions. VCAM-1 and ICAM-1 are involved in the adhesion of cells, while VCAM-1 may play a role in the development of rheumatoid arthritis and atherosclerosis. Moreover, lactoferrin-derived peptides and lactoferrin also showed immunomodulation potentials through regulating antibody production and granulopoiesis. The mechanism by which some of the milk-derived peptides exhibit immunomodulatory attributes has been proposed to be facilitated by the presence of arginine at the *C*- or *N*-terminal [119].

### 5.7. Anti-Cancer

Globally, cancer received much interest as part of the group of life-threatening non-communicable diseases. It is considered one of the topmost causes of death in the 21st century, though more widespread in developed nations compared to developing countries. Various BAPs have been scientifically verified as potential agents for cancer prevention and therapeutic management [120]. Peptides having anti-cancer potential comprise a variation in the structural and geometric configuration and, on their surface, bear the properties related to cationic charges along with hydrophobic parts used to attach to specific cell membrane amphiphatically. Peptides’ hydrophobicity and charge are primarily involved in their functionality against anti-cancer cells [121]. Some mechanisms have been reported for peptides functioning as anti-cancer agents, i.e., initiation of necrosis or apoptosis, angiogenesis process inhibition of cancer cells, immunity boost against tumor cells, retardation of enzymatic activities associated with cancer growth, and distortion of essential proteins related to the proliferation of cancer cells [122]. A previous review reported that the anti-cancer potential of camel milk is fairly higher than that of bovine milk [123]. Moreover, a recent study reported the anti-cancer potential of lactoferrin-derived (LF) peptides. Results confirmed that LFcinB bovine-derived peptide (RRWQWR) showed significant anti-cancer capacity on Jurkat T-leukemia cells [124]. A previous study evaluated the anti-cancer attributes of milk-derived anti-cancer fusion peptide (ACFP) using human ovarian cancer cells [125]. The study collected fresh ovarian tumor tissues from 53 patients and cultured them to produce primary cell lines. Results showed that ACFP treatment hindered viability of the primary ovarian cancer cells through enhanced apoptosis but with no or minimal cytotoxicity against normal ovarian cancer cells [125]. In another study by Rafiq et al. [126], water-soluble peptides (WSP) were extracted from cow and buffalo milk cheddar cheeses and further evaluated for anti-cancer attributes using colon cancer model (HT-29) cells. Results indicated that at 400 and 500 μg WSP/mL concentrations, the maximum inhibition of HT-29 growth was achieved. Parodi’s [127] study reported that cysteine and cysteine-enriched proteins and peptides or c-glutamylcysteine dipeptides could help suppress tumor growth/genesis. Thus, the anti-tumoric property of cheese extracts may be due to the active components present in the cheese.

### 5.8. Anti-Diabetic

Universally, a significant ongoing public health problem has been caused by the increasing incidence of diabetes along with major negative social and economic consequences [128]. Diabetes is mainly associated with increased blood glucose levels and inadequate energy derivation from food consumed. The body’s average blood glucose levels, i.e., 4–6 mM, are regulated through the pancreas’s proper secretion of glucagon and insulin [129]. Several artificial/synthetic medicines are available in the market to mitigate blood sugar or diabetes. However, these drugs have adverse side effects on different body organs, such as high blood sugar and liver damage. Therefore, more researchers are investigating natural remedies or solutions such as BAPs due to their low/negligible side effects. BAPs regulate blood glucose levels through several mechanisms, including inhibition of specific enzymes, especially dipeptidyl peptidase-4 (DPP-IV), α-glucosidase, and α-amylase, as well as acting as receptor agonists of glucagon-like peptide-1 (GLP-1) [130]. Other possible factors through which BAPs regulate diabetes are altering glucose absorption and metabolism, stimulating insulin secretion by AAs that are released during peptide generation, enhancing gut hormone (cholecystokinin, glucagon-like peptide 1, etc.) signaling, and improving uptake of glucose by stimulating PI3K/Akt and AMPK pathways [131]. DPP-IV is found in cell membrane and blood, and this enzyme is responsible for incretin degradation. Hence, DPP-IV inhibition improves the incretin hormone half-life, which leads to enhanced glucoregulatory and insulinotropic capacity [11,132]. Previous evidence has established the DPP-IV inhibitory potentials of peptides isolated from goat casein hydrolysate. In the study, goat casein was hydrolyzed with trypsin/chymotrypsin and further fractionated using two-dimensional silica thin layer chromatography (2D-TLC), followed by LC-MS/MS analysis of the amino acid sequence of peptides [133]. Findings from the study reported five DPP-IV inhibitory peptides (AWPQYL, INNQFLPYPY, VMFPPQSVL, SPTVMFPPQSVL, and MHQPPQPL). From these peptides, INNQFLPYPY was the most active enzyme inhibitor with a DPP-IV IC50 value of 40.08 mM. Moreover, camel milk proteins have been recently used to generate peptides with DPP-IV inhibiting properties. Camel whey protein concentrate was prepared and hydrolyzed into fifteen hydrolysate fractions using trypsin. Results found that fractions H6 and H8 possessed DPP-IV inhibitory IC50 values of 1.52 ± 0.16 and 0.55 ± 0.05 mg/L, respectively. Furthermore, three β-casein-derived peptides, VPF, YPI, and VPV, had DPP-IV IC50 values of 55.1 ± 5.8, 35.0 ± 2.0, and 6.6 ± 0.5 µM, respectively [134]. Another recent study by Jia et al. [135] confirmed the discovery of LDQWLCEKL from α-lactalbumin with DPP-IVIC50 value of 131 µM. Moreover, a previous study reported the anti-diabetic attributes of milk protein and milk protein hydrolysate using type 2 diabetic rats. Results found that after six weeks of feeding (milk protein or milk protein hydrolysate), the diabetic rats had reduced levels of plasma glucose, very-low-density lipoprotein (VLDL), low-density lipoprotein (LDL), total cholesterol, triglycerides, and total blood plasma lipids [136]. Inhibition of α-glucosidase and α-amylase enzymes also contributes to reducing carbohydrates in the blood. These enzymes are responsible for converting complex carbohydrates into simple sugars and are frequently present in intestinal surface cells. α-amylase enhanced the glucose level in the blood by converting glycogen and dietary starch into maltose and glucose. Similarly, α-glucosidase increases the glucose level by breaking disaccharides into glucose in the small intestine [137]. Another study reported the anti-diabetic attributes of peptides derived from camel milk proteins. Results found that WNWGWLLWQL and DNLMPQFM inhibited DPP-IV activity, while MPSKPPLL and KDLWDDFKGL were identified as the most potent inhibitors of porcine pancreatic α-amylase [138]. Table 5 shows the health-promoting and therapeutic attributes of milk-derived bioactive peptides.

## 6. Products from Dairy Peptides

Milk and its products have been used for a long time due to their immense health potential, nutritional value, and long history of safety. Milk is the infant’s primary food and is also considered a significant nutrient source for a regular daily diet. In addition, it is also known for its potential bioactive components such as proteins, peptides, etc. [150]. BAPs are the current area of research that is continuously increasing due to their extended health attributes. Dairy products, cheese, and bovine milk are some of the most prominent food sources used to produce BAPs and bioactive proteins [74]. BAPs are often used as constituents of particular nutritive foodstuffs, like infant formulas and functional foods [151]. These peptides may also be released during the milk product production process. Hypoallergenic infant formulae contain a rich amount of peptides which is formed by the use of hydrolyzed milk proteins [152]. BAPs derived from dairy foods are also available in the commercial market, which claims antihypertensive attributes such as Biozate, Evolus, Calpis, etc., which are majorly produced from casein. BAPs could be used to make functional food ingredients, medicinal products, dietary supplements, and nutraceuticals, and to develop new products to prove health benefits. In addition to that, BAPs could be a good option for establishing personalized nutrition [153]. Although, BAPs from different sources such as plants, meat, edible seeds, etc., show several promising attributes in relation to several disorders such as cancer, diabetes, brain health, hypertension, etc. [7,154,155]. Table 6 represents the commercial dairy products containing peptides that potentially reduce hypertension. 

## 7. Safety of BAPs

BAPs are growing much researcher interest due to their immense nutritional and therapeutic characteristics. Despite their remarkable health-enhancing potential, there is a question about their safety. The main point is that these BAPs are primarily produced using food-grade enzymes, but their safety is not proven scientifically. Several methods are used for the production of the peptides other than the digestive enzymes-based method, such as using several enzymes, using existing or new cultures to new substrates, or new methods, which are not subsisted for human digestion. Consequently, many peptides are deliberated new to people and do not have any safe history of their use, even though they are derivatives of food proteins. So, it is not very clear about their health concerns; newly prepared peptides may contain sequences that cause toxicity or allergic issues [159]. Several components are formed during the processing of peptides or proteins, which may cause severe health concerns in humans, such as allergic constituents, lysinoalanine, D-amino acids, and biogenic amines [160]. In silico study predicted that several AAs such as P, N, H, C or motifs like CYCR, KWK, KKLL, LKL, FKK are commonly revealed in toxic peptides; however, I, K, L, and R, are approved minimally [161,162]. The only method used to form hypoallergenic milk formulas is enzymatic hydrolysis [163,164]. It was reported that formula stimulates the reaction in infants who were allergic to cow milk due to residual peptides [165,166]. Moreover, VRTPEVDDEAL, GAQEQNQEQPIRCELDERF, NSAEPEQSLAC and allergic peptides, were found in hypoallergenic formula [167,168]. However, due to their ample health-promoting attributes, BAPs may be a new point of attraction to making functional foods’ ingredients or nutraceuticals, and many of them have been on the market. Although, there is a lack of BAPs safety studies, which is essential for their commercialization or before their incorporation into food products. Consequently, it must be considered that the duration of peptide use, frequency, and supplementation doses are very useful in evaluating BAPs containing product safety.

## 8. Conclusions and Future Perspectives

Milk proteins are encrypted with several peptides that have potential beneficial activities, especially against human chronic diseases. These peptides can be released through enzymatic hydrolysis of the parent milk proteins and collected for use in the formulation of nutraceuticals and functional foods aimed at health promotion, and also for reducing the overreliance on drugs in combatting diseases. Food-derived peptides are attractive and gaining traction within the scientific community because they contain natural amino acids that do not exhibit the same level of toxicity or side effects as many traditional chemotherapeutics (drugs). However, despite the many benefits of bioactive peptide-based therapies, their wide application in functional foods and nutraceuticals for healthcare delivery has been delayed due to several factors [169]. For example, limited efforts have been made to explore therapeutic or functional foods at the industrial scale due to the lack of advanced technologies for production, structural identification, and product enrichment. Moreover, commercializing BAPs with specific health claims requires studies of their allergenicity, efficacy, and toxicity profiles at different experimental levels such as in silico, in vitro, ex vivo, and in vivo [170]. Despite these limitations, milk protein-derived peptides have great promise as therapeutic agents because of their proven efficacy in ameliorating various metabolic disorders.

## Figures and Tables

**Table 1:** Composition of human and animal milk.

Species	Energy (KJ/Kg)	Ash	Fat	Proteins	Lactose	Dry Matter	Water	References
		Percent						
**Camel**	2745.80	0.85	1.80	1.80	2.91	11.30	90.60	[17,18,19,20]
**Cow**	2983.00	0.78	3.46	3.43	4.71	12.38	87.62	
**Donkey**	1939.40	0.43	1.21	1.74	6.23	9.61	90.39	
**Goat**	3399.50	0.73	4.62	3.41	4.47	13.23	86.77	
**Human**	2855.60	0.22	3.38	1.64	6.69	12.43	87.57	

**Table 2:** Major proteins present in the milk of different species.

Species	Protein	Total Casein	αS1-Casein	αS2 Casein	κ-Casein	β-Casein	Total Whey	α-Lactalbumin	β-Lactoglobulin	References
	(g/L)									
**Camel**	25–45	26.4	5	2.2	0.8	12.8	6.6	3.5	-	[17,20,23,24]
**Cow**	31–38	27.2	10–15	3–4	3–4	9–11	4.5	1–1.5	3.3–4	
**Donkey**	13–28	27.2	0.2–1	0.2	-	3.9	7.5	1.8–3	3.2–3.7	
**Goat**	25–39	25	0–7	4.2	4–4.6	11–18	6	1.2	2.1	
**Human**	9–17	5.6	0.3–0.8	-	0.6–1	1.8–4	8	1.9–2.6	-	

**Table 3:** Milk-derived peptides produced using fermentation.

Source	Sequence/Peptide/Fragment	Fermenting Microorganisms	References
Colostrum powder (bovine)	Peptides lower than 10 kDa MW (P1 and P2 fractions)	*Candida lipolytica*	[39]
Milk	LPYPY peptide	*Lactobacillus delbrueckii*	[41]
Casein protein	DELQDKIHPF peptide	*Lactobacillus helveticus*	[43]
Milk (bovine)	MKLFVPALLSLGALGLCLAA peptide	*Lactobacillus fermentum*	[45]
Milk (camel)	MVPYPQR peptide	*Leuconostoc lactis*	[46]
Whey protein	Peptides lower than (<7 kDa)	*Pediococcus acidilactici*	[47]

**Table 4:** Milk-derived peptides produced using enzymatic hydrolysis methods.

Source	Sequence/Peptide/Fragment	Enzymes Used	References
Whey protein (bovine colostrum)	Three fractions obtained having >30, 10 to 30 and <10 kDa MW	Pepsin and pancreatin	[53]
Buffalo casein (CB)	Highest degree of hydrolysis obtained in molecular weights <3.5 kDa using alcalase	Alcalase, trypsin, pepsin, or papain.	[52]
Buffalo casein hydrolysates (BCH)	RELEE, MEDNKQ, and TVA, EQL peptides	Trypsin and alcalase	[54]
Milk casein (buffalo)	VLPVPQK peptide	Pepsin, trypsin, chymotrypsin	[55]
Skimmed milk (buffalo)	PGPIPK, IPPK, IVPN, and QPPQ peptides	Papain, pepsin or trypsin	[56]

**Table 5:** Health-promoting and therapeutic attributes of milk-derived bioactive peptides.

Source	Peptide Sequence/Fragment	Model/Method Used	Potential Attributes	References
Casein-derived	VPP and IPP	THP-1 human monocytic cell line	Immunomodulatory effect	[38]
Milk	VLPVPQK/PepC	Rat osteoblast cultures	Anti-osteoporotic effect	[79]
Whey-derived	YVEEL and YLLF	Ovariectomized (OVX) osteoporotic rat model	Anti-osteoporotic effect	[80]
Bovine milk	VLPVPQ and VAPFPE	Molecular docking	Anti-hypertensive effect	[84]
Buffalo milk casein	VLPVPQK	In vitro methods	Anti-hypertensive effect	[85]
Goat milk protein	WY	In vitro methods	Anti-hypertensive effect	[87]
Goat milk	Casein fraction	Hypercholesterolaemic rats	Hypocholesterolemic effect	[92]
Bovine milk β-lactoglobulin	IIAEK	Male rats (Wistar strain)	Hypocholesterolemic effect	[94]
Bovine milk	Lactostatin or IIAEK	HepG2, a human liver cell line.	Hypocholesterolemic effect	[93]
Casein-derived	VLPVPQK	Rat osteoblastic cells	Anti-oxidative effect	[101]
Buffalo casein-derived	YFYPQL	In vitro Caco-2 cell model	Anti-oxidative effect	[104]
Buffalo casein-derived	YFYPQL	Mice splenocytes culture	Anti-inflammatory effects	[104]
Milk-derived	RHPHPHLSFM, VPYPQR, HPHPHLSFM, YVPR	In vitro Caco-2 cell model	Anti-oxidative effect	[105]
Camel milk	Peptidoglycan recognition proteins PGRPs (PGRP), lactoferrin	Micro broth dilution assay (in vitro)	Anti-microbial effect	[110]
Camel milk	Whey hydrolysate	Biofilm inhibition, disc diffusion assay, biofilm reduction assay	Anti-microbial effect	[112]
Milk	Milk-derived hydrolysate	Endothelial cells	Immunomodulatory effect	[118]
Bovine milk protein	Anti-cancer fusion peptide (ACFP)	Ovarian cancer cells	Anti-cancerous effect	[125]
Buffalo and cow milk cheddar cheeses	Water-soluble peptide (WSP) extracts	Colon cancer model (HT-29) cells	Anti-cancerous effect	[126]
Goat milk casein	INNQFLPYPY	In vitro assay (DPP-IV-inhibitory activity)	Anti-diabetic effect	[133]
Camel milk proteins	VPV, YPI, and VPF	In vitro assay (DPP-IV-inhibitory activity)	Anti-diabetic effect	[134]
Milk	Milk protein hydrolysate	Diabetic rat	Anti-diabetic effect	[136]
Camel milk protein	KDLWDDFKGL, MPSKPPLL	In vitro assay (DPP-IV-inhibitory activity, porcine pancreatic α-amylase)	Anti-diabetic effect	[138]
Cheddar cheeses (cow and buffalo milk)	Water-soluble peptide (WSP) extracts	Lung cancer (H-1299) cell line	Anti-cancerous effect	[139]
Milk	IPP and VPP	Vascular smooth muscle cells	Anti-hypertensive effect	[140]
Milk (casein hydrolysate)	IPP and VPP	25 male subjects (low hypertension)	Anti-hypertensive effect	[141]
Milk	IPP and VPP	Spontaneously hypertensive rats (SHRs)	Anti-hypertensive effect	[142]
Milk (α-lactalbumin)	STEYG	Mice	Improve bone health	[143]
Goat milk casein	QEPVLGPVRGPFP, SLSSSEESITH, NPWDQVKR, and SDIPNPIGSE	Insulin-resistant HepG2 cells	Anti-diabetic effect	[144]
Casein hydrolysate	VPP and IPP	48 subjects	Anti-hypertensive effect	[145]
Milk	Yogurts containing IPP and VPP	64subjects (men and women)	Anti-hypertensive effect	[146]
Milk	Casein hydrolysate (VPP and IPP)	70 subjects (men and women)	Anti-hypertensive effect	[147]
*L. helveticus* fermented milk	VPP and IPP	94 subjects (men and women) hypertensive	Anti-hypertensive effect	[148]
Milk	Hydrolyzed whey peptide	76 consecutive adult patients (underwent living-donor liver transplantation)	Reduce post-transplant hyperglycemia	[149]

**Table 6:** Dairy products that contain peptides have the potential to reduce hypertension.

Product Name	Protein Source	Processing Method	Peptide	Company	References
Evolus^®^	Casein	Fermentation	IPP, VPP	Valio, Helsinki, Finland	[150,156,157,158]
BioZate^®^	Whey proteins	Hydrolysis with trypsin	Whey peptides	Davisco, Minnesota, USA	
Calpis^®^	Casein	Fermentation	IPP, VPP	Calpis Co., Tokyo, Japan	
Danaten^®^		Fermentation	ND	Danone, Paris, France	
Ameal S^®^	Casein	Fermentation	IPP, VPP	Calpis Co., Tokyo, Japan	
C^12^ peptide^®^	Casein	Hydrolysis with trypsin	FFVAPFPEVFGK	DMV International, Holland, Netherlands	

ND: Not described.

## Data Availability

Not applicable.
